# Identification of LncRNA Prognostic Signature Associated With Genomic Instability in Pancreatic Adenocarcinoma

**DOI:** 10.3389/fonc.2022.799475

**Published:** 2022-03-29

**Authors:** Jinfeng Zhu, Qian Huang, Xingyu Peng, Chen Luo, Sicheng Liu, Zitao Liu, Xun Wu, Hongliang Luo

**Affiliations:** ^1^ Department of General Surgery, The Second Affiliated Hospital of Nanchang University, Nanchang, China; ^2^ Jiangxi Province Key Laboratory of Molecular Medicine, Nanchang, China; ^3^ Department of General Practice, The Third Xiangya Hospital, Central South University, Changsha, China

**Keywords:** genomic instability, long non-coding RNA, signature, pancreatic adenocarcinoma, prognosis, chemosensitivity

## Abstract

**Background:**

Genomic instability (GI) is a critical feature of cancer which plays a key role in the occurrence and development of pancreatic adenocarcinoma (PAAD). Long non-coding RNA (LncRNA) is an emerging prognostic biomarker because it is involved in regulating GI. Recently, researchers used such GI-related LncRNAs (GILncRNAs) to establish a prognostic signature for patients with cancer and helped in predicting the overall prognosis of the patients. However, it is evident that patients with PAAD still lack such prognostic signature constructed with GILncRNA.

**Methods:**

The present study screened GILncRNAs from 83 patients with PAAD. Prognosis-related GILncRNAs were identified by univariate Cox regression analysis. The correlation coefficients of these GILncRNAs were obtained by multivariate Cox regression analysis and used to construct a signature. The signature in the present study was then assessed through survival analysis, mutation correlation analysis, independent prognostic analysis, and clinical stratification analysis in the training set and validated in the testing as well as all TCGA set. The current study performed external clinical relevance validation of the signature and validated the effect of AC108134.2 in GILncSig on PAAD using *in vitro* experiments. Finally, the function of GILncRNA signature (GILncSig) dependent on Gene Ontology enrichment analysis was explored and chemotherapeutic drug sensitivity analysis was also performed.

**Results:**

Results of the present study found that a total of 409 GILncRNAs were identified, 5 of which constituted the prognostic risk signature in this study, namely, AC095057.3, AC108134.2, AC124798.1, AL606834.1, and AC104695.4. It was found that the signature of the present study was better than others in predicting the overall survival and applied to patients with PAAD of all ages, genders, and tumor grades. Further, it was noted that the signature of the current study in the GSE102238, was correlated with tumor length, and tumor stage of patients with PAAD. *In vitro*, functional experiments were used in the present study to validate that AC108134.2 is associated with PAAD genomic instability and progression. Notably, results of the pRRophetic analysis in the current study showed that the high-risk group possessed reverse characteristics and was sensitive to chemotherapy.

**Conclusions:**

In conclusion, it was evident that the GILncSig used in the present study has good prognostic performance. Therefore, the signature may become a potential sensitive biological indicator of PAAD chemotherapy, which may help in clinical decision-making and management of patients with cancer.

## Introduction

Pancreatic adenocarcinoma (PAAD) is one of the most deadly malignant tumors, which is ranked fourth for cancer-related deaths in the United States (1). Previous studies have predicted that PAAD will soon overtake breast cancer and become the third leading cause of cancer death in the European Union (2) in 2025. Therefore, there is an urgent need to find therapeutic targets and construct prognostic signature for patients with PAAD.

Genomic instability (GI) is an important feature of cancer (3), which plays a key role in the occurrence and development of PAAD. Telomere fusion and damage (4–6), centrosome amplification (7), epigenetic modification (8–10), mitochondrial DNA changes (11, 12), and DNA damage (13) among others can destroy the stability of the genome and induce occurrence of tumors.

Furthermore, GI affects the progression of PAAD at multiple genetic levels. For instance, at the genetic level a global study on PAAD genome sequencing indicated that most patients had gene point mutations and deletions, and a few had gene amplification (14). Moreover, the loss of SMAD4 promoted KRAS (G12D)-mediated metastasis of PAAD (15). On the other hand, at the chromosomal level, previous studies have shown that loss of chromosome 18q was an important manifestation of early PAAD (16). In addition, it has been found that the targeting P15 (INK4b) promoter of a nuclear factor of activated T cell (NFAT)c2 promoted the growth of PAAD by inducing heterochromatin protein HP1γ (17).

Therefore, further research on GI is of great significance for the treatment of PAAD. Recently, it has been reported that the GI-related lncRNA signature can predict the prognosis of malignant tumors. For instance, some studies have pointed out that GI can be used as a biomarker for poor prognosis in patients with breast cancer (18), prolactinoma (19), and renal clear cell carcinoma (20) among others. Furthermore, it has been reported that increased levels of GI is associated with a higher risk of death (21). However, there is still a lack of GI-related lncRNA prognostic signature in pancreatic cancer.

Long non-coding RNA (LncRNA) is a kind of RNA with more than 200 nucleotides in length and without protein-coding ability. It regulates gene expression at the epigenetic, transcriptional, and translational levels primarily through interactions with RNA, DNA, or proteins (22). Recently, several studies have confirmed that LncRNA can effectively maintain the stability of the genome (23, 24). The possible mechanism of this phenomenon is that LncRNA can effectively regulate the formation of aneuploidy (25), stabilizes telomere length (26), and participates in the repair of DNA double-bond fracture (27).

Therefore, several researchers set out to prognostic risk signature for cancer patients using GI-related LncRNA signature (GILncSig), to help assess the overall prognosis of patients with cancer. For instance, a study conducted by Geng et al. (28) constructed a prognostic signature for patients with lung adenocarcinoma (LINC01133, LINC01116, LINC01671, FAM83A-AS1, PLAC4, MIR223HG, and AL590226.1). Elsewhere, Yang et al. (29) created a prognostic signature of renal clear cell carcinoma composed of 7-LncRNA (LINC00460, AL139351.1, AC156455.1, AL035446.1, LINC02471, AC022509.2, and LINC01606). Furthermore, the studies of Wu et al. (30), Yin et al. (31), Maimaiti et al. (32), and Yan et al. (33) also developed a prognostic signature for patients with bladder cancer, colon cancer, low-grade glioma, and melanoma, respectively.

In the present study, a prognostic risk signature for patients with PAAD was established using GILncRNAs in the TCGA database, to help assess patient survival outcomes and optimize clinical management of cancer.

## Materials and Methods

### Research Process and Data Collection

**Figure 1:**
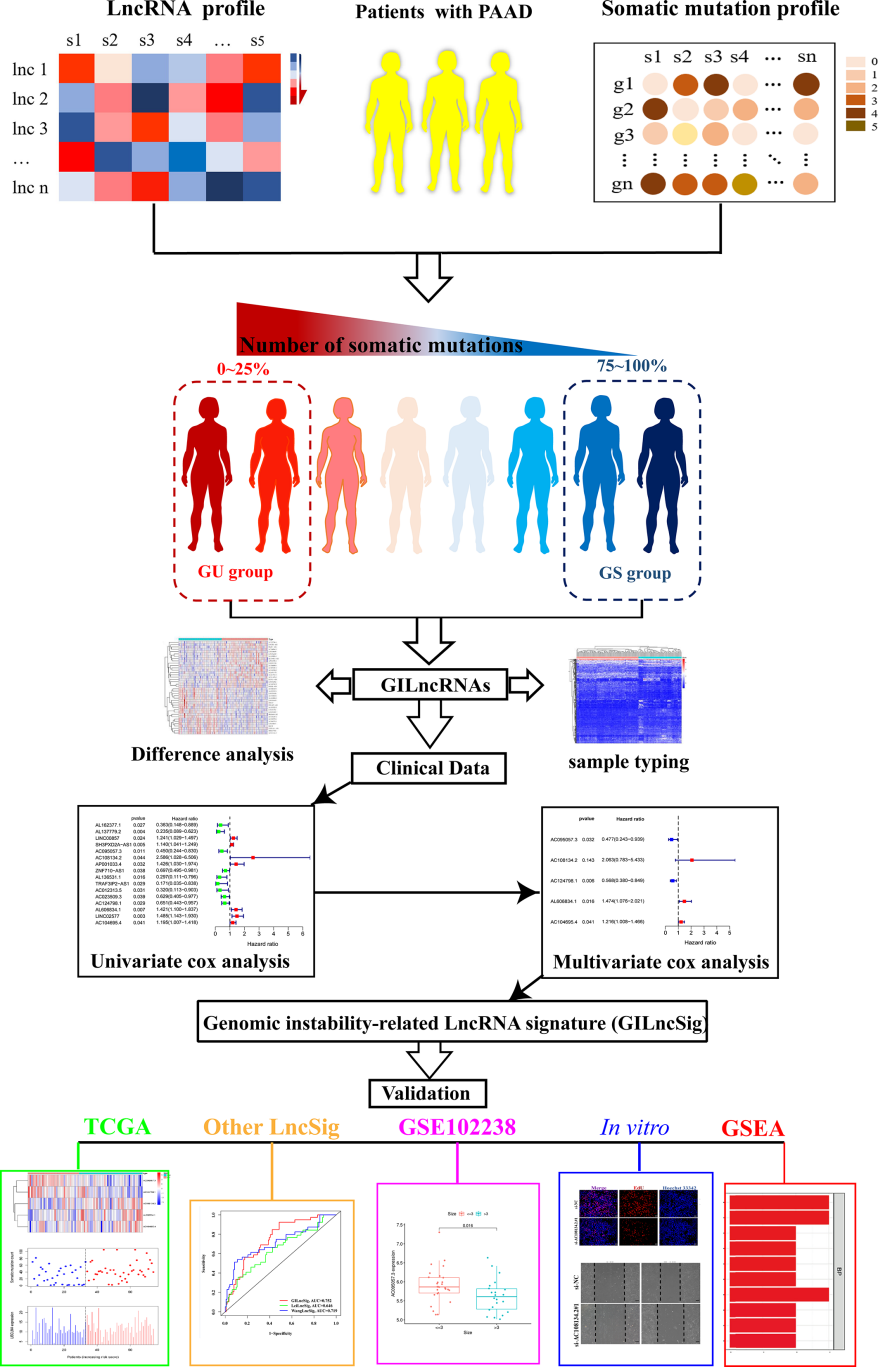
Flow chart of research design used in this article.

The research process of the current study is detailed in **Figure 1**. Transcriptome data of 182 patients with PAAD (normal sample: 4; tumor sample: 178), mutation data of 158 patients with PAAD (VarScan version), and clinical data of 185 patients with PAAD was downloaded from The Cancer Genome Atlas (TCGA) database (https://portal.gdc.cancer.gov/).

The external validation, clinical, gene expression, and gene annotation information of 100 patients with PAAD were downloaded from the gene expression Omnibus (GEO) (34) database (independent dataset GSE102238, GPL19072 platform). Among the downloaded datasets, it was found that dataset GSE102238 contains clinical and gene expression data, whereas the GPL19072 platform only had gene sequences. Therefore, gene sequences were annotated as gene names using the “GEOquery” package of R software to facilitate the analysis in the current study.

### Screening of LncRNAs Associated With Genomic Instability

The number of mutations in each sample was calculated based on the gene mutation data and ranked them in descending order. The top 25% samples were defined with the largest number of mutations as the genomic unstable (GU) group and the bottom 25% samples with the lowest number of mutations as the genomic stable (GS) group. The ID numbers of the GS and GU groups were then matched with gene transcriptome data to obtain LncRNA expression levels of the two groups, respectively. The mean expression of each lncRNA between the GS and GU groups was then compared using the Wilcoxon rank-sum test in the “limma” package of R software. LncRNAs, |logFC| >1 and false discovery rate (FDR) adjusted *p <*0.05, were defined as LncRNAs related to genomic instability (GILncRNAs).

### Sample Classification (Hierarchical Clustering Algorithm)

Expression data of GILncRNAs from 178 PAAD samples was quantized and hierarchical clustering analysis was performed by calculating Euclidean distances with the help of “sparcl” and “limma” packages and cut the tree into two clusters. Those with a high number of mutations were defined as genomic unstable-like (GU-like) group and those with a low number of mutations as genomic stable-like (GS-like) group. The number of the GU-like and GS-like groups of somatic mutation count and the expression level of UBQLN4 (35), a tumor driver gene that inhibited DNA double-strand break (DSB) repair, were then compared to see if the LncRNA in the present study reflected GI.

### Establishment of Prognostic Risk Signature

The samples with no survival information or survival time less than 30 days were deleted and survival data of 171 samples was obtained. Then, after combining the transcriptome data of GILncRNA with survival data, the samples were randomly divided into the training and testing sets. The “survival” package was used to conduct univariate and multivariate Cox regression analysis on the survival data of GILncRNAs in the training set to complete the construction of the prognostic risk signature. The formula for calculating the GILncSig risk score was as follows:


$$GILncSig\;risk\;score=\sum_{i=1}^{n}coef\left(lncRNAi\right)\times exp\left(lncRNAi\right)$$

Where:

“coef (lnRNAi)”, “exp (lncRNAi)”, and “n” represent the coefficient, expression level, and the number of prognostic lncRNAs, respectively. Coef >0 indicated that LncRNA was a risk factor for survival, whereas coef <0 showed that LncRNA was a protective factor for survival.

According to the above formula, the risk values of all samples in the training, testing, and the TCGA sets were hence calculated. The samples above or below the median risk value were respectively divided into the high- or low-risk groups.

### Evaluation and Verification of the Signature Within the TCGA

Chi-square test was performed on the clinicopathological features of the training and testing sets to confirm whether the two groups were comparable.

#### Survival Analysis and Signature Prediction Accuracy Assessment

Survival analyses between the high- and low-risk groups were plotted through “survival” and “survminor” software packages. The “timeROC” package was used to draw the ROC curve at 1-, 3-, 5-year and hence the area under the curve (AUC) was calculated.

#### Mutation Correlation Analysis

First, the risk curve was plotted and the approximate relationship between the values of risk, number of somatic mutation count, and UBQLN4 expression were observed. The “limma” and “ggpubr” packages were used to further validate the previous results. The genes with the highest number of mutations in the study were then selected to compare their mutation percentages in the high- and low-risk groups. Finally, a co-survival analysis of patients with different clusters and different gene states was performed.

#### Independent Prognostic Analysis

Cox regression analysis was conducted for each variable in the training set using the “survival” package to prove if GILncSig was an independent prognostic factor.

#### Clinical Stratification Analysis

The survival rates of patients with PAAD and different clinicopathological features in the high- and low-risk groups were compared through the Wilcoxon test.

### Signature Comparison and External Clinical Correlation Verification

Other prognostic risk signatures for patients with PAAD were collected and verified whether the signature in the current study was better by comparing the AUC. In addition, a correlation analysis was also performed for different clinical characteristics of patients with PAAD in the GEO database.

### Samples

The tissues of patients with PAAD and their adjacent tissues were collected from the Second Affiliated Hospital of Nanchang University. This study was approved by the Medical Ethics Committee of the Second Affiliated Hospital of Nanchang University. The experiment was understood and agreed in writing by each subject and the applied research method met the standards set out in the Declaration of Helsinki.

### Cell Culture and Transfection

The human PAAD cell line (BxPC-3 cell) was purchased from the Cell Bank of the Chinese Academy of Sciences of Shanghai. The cell lines were verified through short tandem repeat (STR) sequence identification of the cell bank. They were cultured in 1640 (Gibco, USA) supplemented with 10% fetal bovine serum and 100 units/ml penicillin–streptomycin (Invitrogen, USA) at 37 °C in a humid atmosphere containing 5% CO2. Further, the AC108134.2 specific siRNA and negative control siRNA were purchased from GenePharma (Shanghai, China) and transfected with Lipofectamine 3000 reagents (Invitrogen, Waltham, USA) transfected BxPC-3 according to standard guidelines. The sequence of siRNA is shown in **Table S2**.

### Quantitative Real-Time (qRT)-PCR

Total RNA was extracted from the collected tissues and cells using Trizol reagent (Invitrogen, USA). qRT-PCR was performed using SYBR Green qPCR Master Mix (Clontech Laboratories, USA) with Applied Biosystems 7900HT Fast Real-Time PCR System (Thermo Fisher Scientific, USA). The primers used in the current study are shown in **Table S2**.

### Cell Counting Kit-8 (CCK-8) Assay and 5-Ethynyl-2’-Deoxyuridine Assay (EdU) Assay

The proliferation ability of BxPC-3 cells with/without AC108134.2 downregulation was observed using CCK-8 assay and EdU staining assay. The control and treated cells were seeded in 96-well plates at 8 × 10^3^ cells per group and incubated until cell attachment occurred. Subsequently, 10 μl of CCK-8 reagent was added to each well at 0, 24, 48, and 72 h, respectively. Further, 2 h after CCK-8 administration, the absorbance value of each well was measured at a wavelength of 450 nm. In addition, both groups of cells were incubated with EdU (50 µM) (Guangzhou RiboBio, China) for 2 h according to the instructions provided by the manufacturer. After fixing and permeabilizing cells, 1 × Apollo Stain Reaction was used for 30 min at room temperature. Nuclear DNA was stained with 1 × Hoechst33342 for half an hour at room temperature. Subsequently, the cells were observed under a fluorescence microscope and photographed for analysis.

### Wound Healing Assay and Transwell Invasion Assay

The cell migration ability was examined using a wound-healing assay. Briefly, two groups of cells were separately seeded in 6-well plates and scraped using a sterile pipette (200 μl) when cells reached approximately 95% confluency. Wound images were then taken with an inverted microscope at 0, 24, and 48 h. Images were analyzed using ImageJ, which can analyze the ability of cells to migrate by calculating wound area. In addition, transwell invasion assays were performed using Transwell chambers with Matrigel (BD Sciences, Sparks, MD, USA). The invasion ability of BxPC-3 cells with downregulated/not downregulated AC108134.2 was observed through transwell assay. Two groups of cells were seeded in transwell chambers, 5 × 10^4^ cells per group. After 24 h, cells were fixed with 4% formaldehyde solution and cells were then stained with 0.2% crystal violet solution for 20 min. Finally, the cells were removed from the inner surface of the cells with a cotton swab and an inverted microscope was used to count invading cells.

### Protein Extraction and Western Blot

The BxPC-3 cells were harvested and their total protein was extracted using RIPA lysis buffer. After protein concentration was determined by BCA, protein loading buffer was added and boiled for 10 min. Proteins were then electrophoresed using 10% SDS-PAGE and transferred to 0.22 μm PVDF membranes and blocked with 5% nonfat milk for 1 h at room temperature. The corresponding primary antibodies were incubated overnight at 4°C and then washed thrice with 1× TBST for 10 min each. The secondary antibody of the corresponding species was incubated for 1 h at room temperature and then washed 3 times. The exposure and photograph were taken using ECL luminescence solution. The data obtained in the present study were organized and analyzed using Image J software. Antibodies and dilution ratios were as follows: anti-MLH1 (CST, # 3515; 1:300), anti-MSH2 (CST, # 2017; 1:1,000), anti-MSH6 (CST, #12988; 1:1,000), anti-GAPDH (Proteintech, 60004-1-Ig; 1:10,000).

### Searching for Possible Functions Related to GILncSig

The 10 mRNAs most related to GILncSig were obtained through Pearson co-expression analysis; a GILncSig-mRNA co-expression network was formed and realized visualization with the “igraph” package. The “clusterProfiler”, “org.Hs.eg.db”, “enrich plot”, and “ggplot2” packages of R software were then used for GO enrichment analysis and bar charts were drawn to show the most relevant 10 functions.

### Relationship Between the Signature and Clinical Treatment

The IC50 values of the commonly used chemotherapeutic drugs were evaluated using high-throughput sequencing data of PAAD in the TCGA to clarify the role of the signature in clinical treatment. The Wilcoxon signed-rank test was used in the present study to compare the differences between the two groups and the visualization of the results was performed using pRRophetic and ggplot2.

### Statistical Analysis

The R-version 3.6.2 was used for statistical analysis in the current study. Comparisons between the two groups were analyzed using the Student’s *t*-test. Survival analysis was completed using the Kaplan–Meier method. Data from at least three separate experiments were presented as means  ±  (SEM). The statistically significant difference was set at *p <*0.05.

## Results

### Identifying Genomic Instability-Related LncRNAs

**Figure 2:**
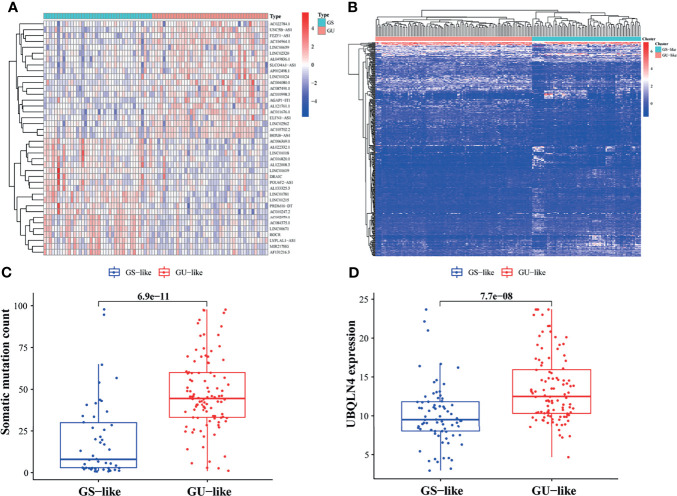
Screening and validation of lncRNAs associated with genomic instability (GILncRNAs). **(A)** GILncRNAs were selected by comparing gene expression levels of the gene stable and gene unstable (GU) groups. Twenty GILncRNAs with the most obvious upregulation and downregulation were separately shown in the heat map. **(B)** 409 GILncRNAs were classified using the clustering algorithm. The genomic stable-like (GS-like) group is depicted in the left red cluster, whereas, the genomic unstable-like (GU-like) group is described in the right blue cluster. **(C)** Comparison of somatic accumulative mutation count between the GS-like and GU-like groups. **(D)** Comparison of UBQLN4 expression level between the GS-like and GU-like groups.

In the Long non-coding RNA (LncRNA) samples of PAAD sorted by the number of mutations in descending order, it was found that 43 were in the GU group whereas 40 were in the GS group. After differential analysis of gene expression levels between the two groups, a total of 409 LncRNAs were found to be significantly differentially expressed, of which 169 were upregulated and 240 downregulated in the GU group (**Table S1**). The GILncRNAs that most significantly upregulated (n = 20) and downregulated (n = 20) were shown in the heat map (**Figure 2A**).

Algorithm clustering was used to classify 178 LncRNA samples and classify them into GU-like group and GS-like group according to mutation frequency (**Figure 2B**). The gene mutation frequency of the two LncRNA clusters was then compared and it was found that the number of GU-like group somatic mutation count was significantly higher than that of the GS-like group (*p <*0.001, **Figure 2C**). Further, the expression level of UBQLN4 was analyzed in different LncRNA clusters and it was observed that the expression level of UBQLN4 was higher in the GU-like group (*p <*0.001, **Figure 2D**). The described results of the present study suggested that these 409 LncRNAs may be associated with GI.

### Establishing a GILncSig

**Figure 3:**
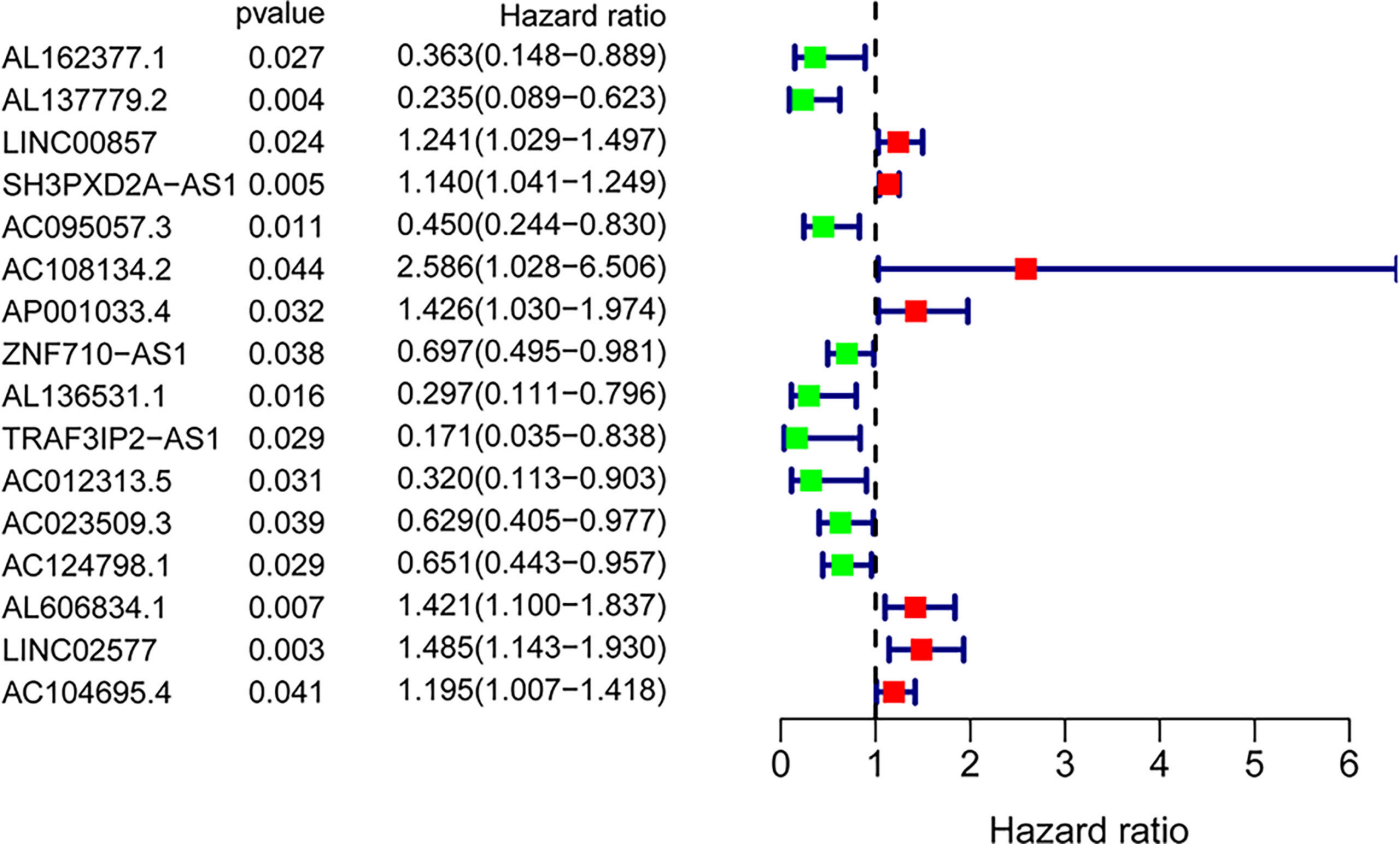
Forest plot of 16 GInLncRNAs associated with overall survival of patients based on univariate Cox regression analysis.

A total of 171 GILncRNA samples were randomly divided into the training (n = 86) and testing (n = 85) sets to establish a prognostic risk signature. Univariate Cox regression analysis was performed on the survival data of the training set, and a total of 16 GILncRNAs were found to be associated with prognosis (**Figure 3**). Multivariate Cox regression analysis was then conducted on these GILncRNAs and established prognostic risk signature composed of 5-GILncRNA (AC108134.2, AL606834.1, AC104695.4, AC095057.3, and AC124798.1) (**Table 1**). It was found that the risk genes for survival of patients with PAAD were AC108134.2, AL606834.1, and AC104695.4, whereas the protective genes were AC095057.3 and AC124798.1. Furthermore, the prognostic risk signature formula was as follows:GILncSig risk score = expAC108134.2 ∗ 0.724 + expAL606834.1 ∗ 0.389 + expAC104695.4 ∗ 0.195 + expAC095057.3 ∗ (−0.740) + expAC124798.1 ∗ (−0.566)

**Table 1:** Long non-coding RNA signature models associated with genomic instability.

GILncSig	Coef	HR	HR (95%CI)	*p*
AC095057.3	−0.740	0.477	0.243–0.939	0.032
AC108134.2	0.724	2.063	0.783–5.433	0.143
AC124798.1	−0.566	0.568	0.380–0.849	0.006
AL606834.1	0.389	1.474	1.076–2.021	0.016
AC104695.4	0.195	1.216	1.008–1.466	0.041

HR, hazard ratio; CI, confidence interval.

### Evaluating and Verificating the Signature in the TCGA

**Figure 4:**
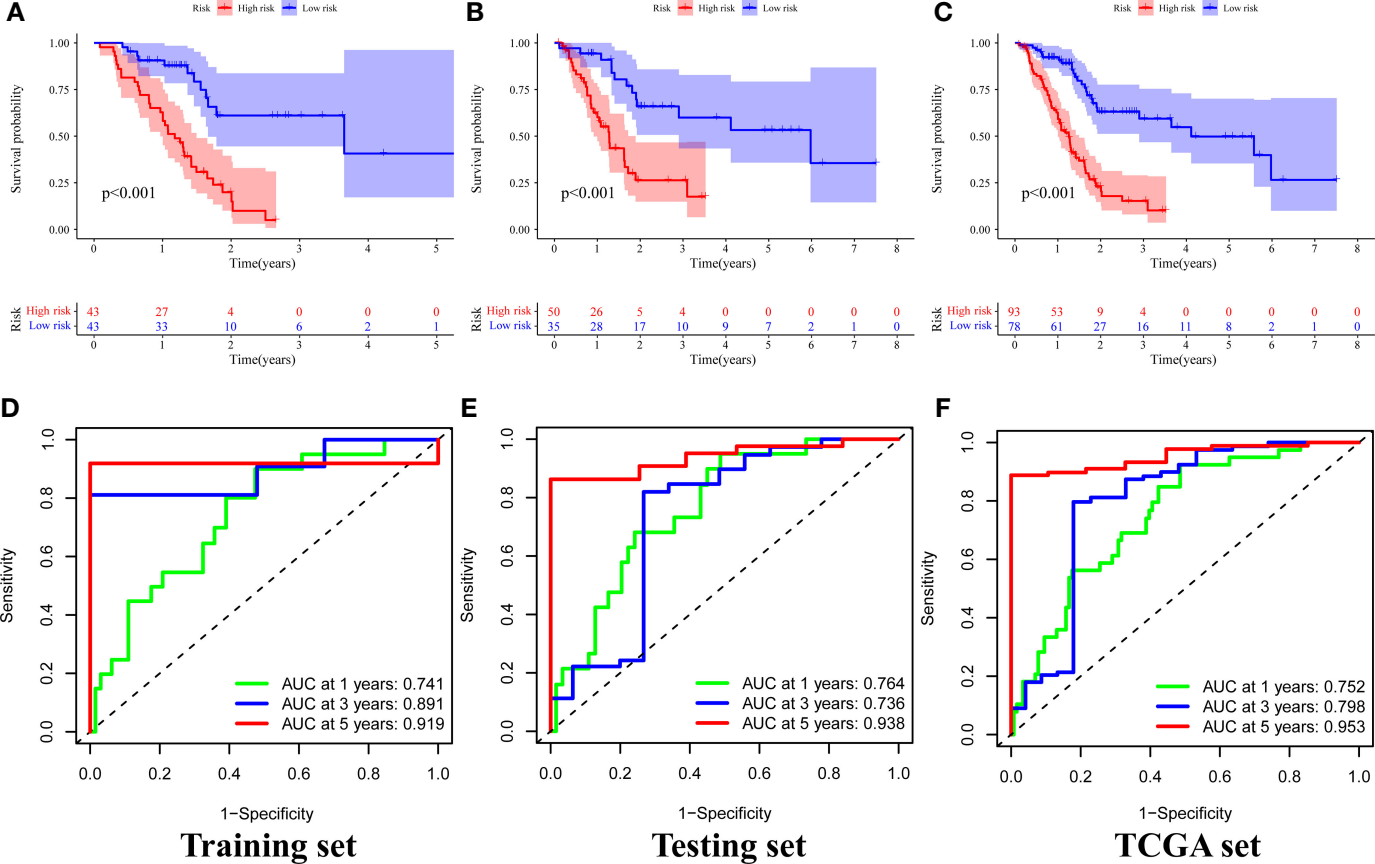
Survival analysis and results of signature prediction accuracy assessment in the training, testing, and TCGA sets. Comparison of survival rates between high-risk and low-risk groups in the training **(A)**, testing **(B)**, and TCGA sets **(C)**. ROC curve for 1-, 3-, and 5-year survival prediction of the signature in the training **(D)**, testing **(E)**, and TCGA **(F)** sets.

A chi-square test was performed on the clinical data of the training and the testing sets before evaluation and validation and it was found that there was no significant difference (all *p >*0.05, **Table 2**). This showed that random grouping in the present study was reasonable. Further, it was evident that the survival analysis in the training set showed that the survival rate in the high-risk group was significantly lower than that in the low-risk group (*p <*0.001, **Figure 4A**). Pleasantly, similar results were also obtained in both the testing (*p <*0.001, **Figure 4B**) and the TCGA sets (*p <*0.001, **Figure 4C**). A ROC curve for 1-, 3-, and 5-year survival prediction of the signature was also drawn and the AUC value was calculated as 0.741, 0.891, and 0.919, respectively in the training set (**Figure 4D**), as 0.764, 0.736, and 0.938, respectively in the testing set (**Figure 4E**), and as 0.752, 0.798, and 0.953 respectively in the TCGA set (**Figure 4F**). Results of the present study indicated that the GILncSig in the present study had a high predictive performance for survival within 5 years in patients with PAAD.

**Table 2:** Comparison of clinicopathological features between the training set and testing set.

Covariates	Type	Total (n = 171)	Training set (n = 86)	Testing set (n = 85)	*p*
Age	≤65	90 (52.63%)	44 (51.16%)	46 (54.12%)	0.815
	>65	81 (47.37%)	42 (48.84%)	39 (45.88%)	
Gender	Female	78 (45.61%)	37 (43.02%)	41 (48.24%)	0.596
	Male	93 (54.39%)	49 (56.98%)	44 (51.76%)	
Grade	G1–2	120 (70.18%)	63 (73.26%)	57 (67.06%)	0.333
	G3–4	49 (28.65%)	21 (24.42%)	28 (32.94%)	
	Unknown	2 (1.17%)	2 (2.33%)	0 (0%)	
Stage	Stage I–II	161 (94.15%)	82 (95.35%)	79 (92.94%)	1.000
	Stage III–IV	7 (4.09%)	4 (4.65%)	3 (3.53%)	
	Unknown	3 (1.75%)	0 (0%)	3 (3.53%)	
T stage	T1–2	28 (16.37%)	17 (19.77%)	11 (12.94%)	0.351
	T3–4	141 (82.46%)	69 (80.23%)	72 (84.71%)	
	Unknown	2 (1.17%)	0 (0%)	2 (2.35%)	
M stage	M0	77 (45.03%)	42 (48.84%)	35 (41.18%)	1.000
	M1	4 (2.34%)	2 (2.33%)	2 (2.35%)	
	Unknown	90 (52.63%)	42 (48.84%)	48 (56.47%)	
N stage	N0	47 (27.49%)	28 (32.56%)	19 (22.35%)	0.200
	N1	119 (69.59%)	56 (65.12%)	63 (74.12%)	
	Unknown	5 (2.92%)	2 (2.33%)	3 (3.53%)	

p < 0.05 means significantly different.

**Figure 5:**
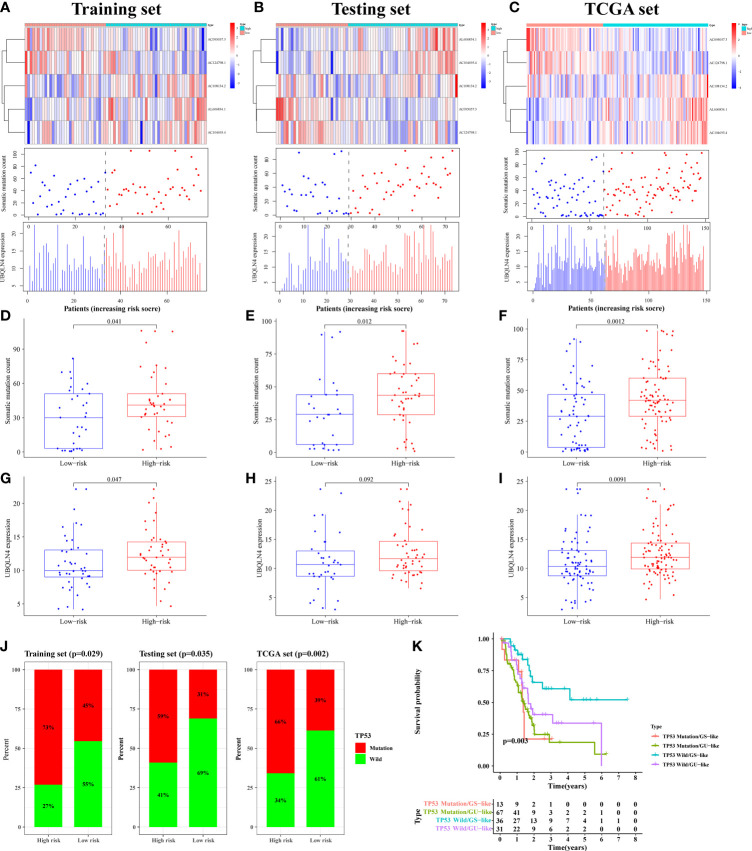
The mutation correlation analysis using for signature verification. The risk curves of the training **(A)**, testing **(B)** and TCGA **(C)** sets were composed of heat map, gene mutation point map, and UBQLN4 expression map. It reflected the changes in the number of gene mutations and the expression levels of UBQLN4 and GILncSig with the increase of risk value. Comparison of somatic mutation count between the high- and low-risk groups in the training **(D)**, testing **(E)** and TCGA **(F)** sets. Comparison of UBQLN4 expression levels between the high- and low-risk groups in the training **(G)**, testing **(H)** and TCGA **(I)** sets. **(J)** Comparison of the proportion of TP53 mutation status between the high- and low-risk groups in the training, testing and TCGA sets. **(K)** Combined survival analysis curve graph based on GILncSig clustering and TP53 mutation status.

By plotting the risk curve, we found that the number of mutations in the training, testing and TCGA sets all showed an increasing trend with the increase of risk value (**Figures 5A–C**). Genes with high expression levels in the high-risk group were AC108134.2, AL606834.1, AC104695.4, and UBQLN4 (**Figures 5A–C**). To further verify the above relationship, we compared the number of somatic mutation and UBQLN4 expression levels between the high- and low-risk groups. In the training set, we observed that the number of somatic mutation in the high-risk group was significantly higher than that in the low-risk group (p = 0.041, **Figure 5D**). Similarly, in the testing (p = 0.012, **Figure 5E**) and TCGA (p = 0.0012, **Figure 5F**) sets, the high-risk group had more somatic mutation count. UBQLN4 expression level in the high-risk group was higher than that in the low-risk group, which was shown in the training (p = 0.047), testing (p = 0.092), and TCGA (p = 0.0091) sets (**Figures 5G–I**).

The LncRNA had two states (wild state and mutant state) and hence the signature was verified by comparing the proportion of single gene mutation state. In the current study, the TP53 (36), the driver gene of PAAD with high mutation frequency, was selected as the validation object. Through analysis, it was found that the proportion of TP53 mutation status in the training, testing, and TCGA sets, of the high-risk group were all significantly higher than that in the low-risk group (*p* < 0.05, **Figure 5J**).

The pairs of samples from different states (wild and mutant) and clusters (GS-like and GU-like) were combined for combined survival analysis. It was revealed that the survival rates of TP53 mutation/GS sample (n = 13), TP53 mutation/GU sample (n = 67), TP53 wild/GS sample (n = 36), and TP53 wild/GU sample (n = 31) were significantly different (*p* = 0.003, **Figure 5K**). Further, it was found that the survival rate of patients with PAAD with TP53 mutation decreased rapidly to 50% within one and a half years of onset, whereas the survival rate of patients with PAAD with TP53 wild/GS-like remained above 50% after 4 years of onset. Therefore the results of the present study suggested that the developed prognostic risk signature can well predicted gene mutation frequency and survival in patients with PAAD.

Subsequently, an independent prognostic analysis was performed on the signature to verify whether GILncSig was an independent prognostic factor that was immune to clinical factors. Univariate COX analysis was also conducted on clinicopathological features (age, sex, tumor grade, and tumor stage) as well as the risk values of patients in the training set and it was evidently found that the age (*p* = 0.009) and risk value (*p* = 4.35 × 10^−6^) may be independent of other clinical factors (**Table 3**). Multivariate Cox regression for these two factors was also performed and it was confirmed that the prognostic risk signature (*p* = 6.25 × 10^−6^) and the age (*p* = 0.010) were the independent prognostic factors for patients with PAAD (**Table 3**).

**Table 3:** Independent prognostic analysis results of GILncSig in the training set.

Variables		Univariate Cox regression analysis			Multivariate Cox regression analysis		
		HR	95%CI	*p*	HR	95%CI	*p*
Training set (n = 86)							
Age		1.043	1.011–1.076	0.009	1.044	1.010–1.078	0.010
Gender	Female/Male	0.895	0.497–1.612	0.712			
Grade	1/2/3/4	1.439	0.955–2.168	0.082			
Stage	I/II/III/IV	1.253	0.761–2.064	0.375			
Risk score	High/Low	1.324	1.175–1.492	<0.001	1.324	1.172–1.495	<0.001

HR, hazard ratio; CI, confidence interval.

**Figure 6:**
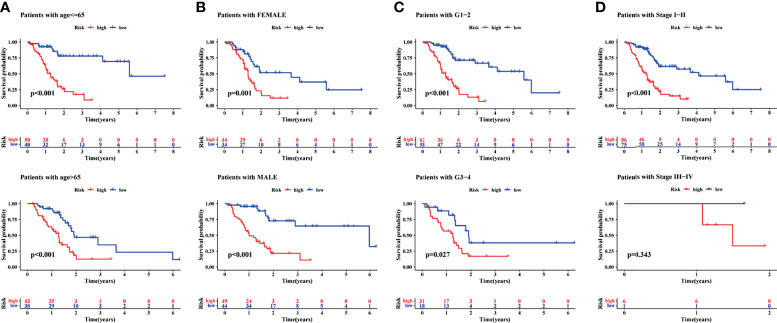
A clinical stratified analysis of survival including age, sex, tumor grade, and tumor stage. Comparison of survival rate between high- and low-risk groups of patients with age ≤65 years or >65 years **(A)**, the gender of female or male **(B)**, the tumor grade of G1–2 or G3–4 **(C)**, the tumor stage of I–II or III–IV **(D)**.

Finally, to understand the applicability of the signature, the signature was used to finish a clinicopathological stratified analysis of differences in survival, namely, age, sex, tumor grade, and tumor stage. Results of the current study showed that the survival rate was significantly lower in the high-risk group than in the low-risk group when patients with age younger than (*p <*0.001) or older than 65 years (*p <*0.001, **Figure 6A**), the gender of male (*p <*0.001) or female (*p* = 0.001, **Figure 6B**), the tumor grade of 1–2 (*p <*0.001) or 3–4 (*p* = 0.027, **Figure 6C**), and the tumor stage of I–II (*p <*0.001, **Figure 6D**). Results of the present study suggested that the signature was applied to patients with early PAAD of any age, sex, and tumor grade.

### Signature Comparison and External Verification

**Figure 7:**
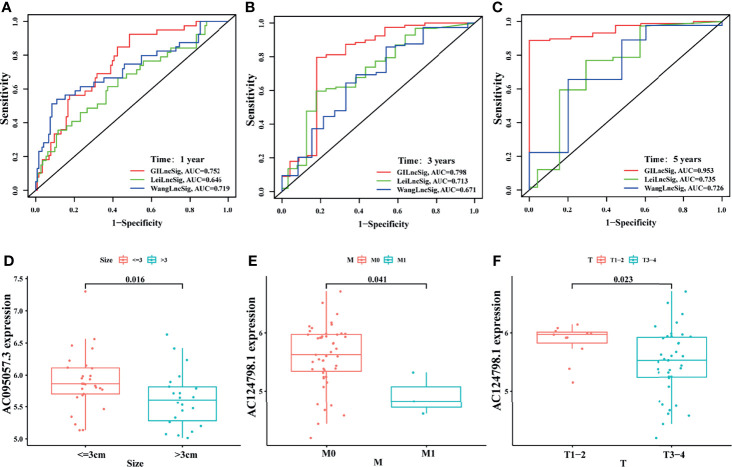
Comparison and external verification of the signature. The ROC analyses of overall survival at 1- **(A)**, 3- **(B)**, and 5- **(C)** year for the GILncSig, LeiLncSig, and WangLncSig. Relationship between AC095057.3 and tumor size **(D)**; between AC124798.1 and M stage **(E)**, and T stage **(F)** in GSE102238 cohort.

To highlight the advantages of the signature, it was compared with other models that had been established. One was the irLncRNAs model (LINC00462, LINC01887, RP11-706C16.8) established by Lei et al. (37), the other was the 7-LncRNAs model (LINC00941, UNC5B-AS1, AL049555.1, MIR600HG, CASC8, AL365277.1, AC005056.1) established by Wang et al. (38). By plotting the ROC curve, it was found that the area under the ROC curve of the current signature for 1-, 3-, and 5-year survival prediction was all the largest (AUC = 0.752, 0.798, and 0.953, respectively), which was significantly greater than those established by Lei (AUC = 0.646, 0.713, and 0.735) and Wang (AUC = 0.719, 0.671, and 0.726) (**Figures 7A–C**). This indicated that the predictive performance of the current signature for prognosis was better than some existing signatures.

In addition, some clinicopathological features of patients with PAAD in the GEO dataset (GSE102238) were correlated with the current signature. The present study revealed that the expression level of protective gene AC095057.3 was higher in tumors less than 3 cm in size (*p* = 0.016, **Figure 7D**). In addition, the expression of protective gene AC124798.1 was higher in M0 (*p* = 0.041, **Figure 7E**) and T1–2 (*p* = 0.023, **Figure 7F**) than in M1 and T3–4. Results of this study indicate that the currently established signatures are partially verified in the GSE102238 dataset.

### Experimental Validation *In Vitro*

**Figure 8:**
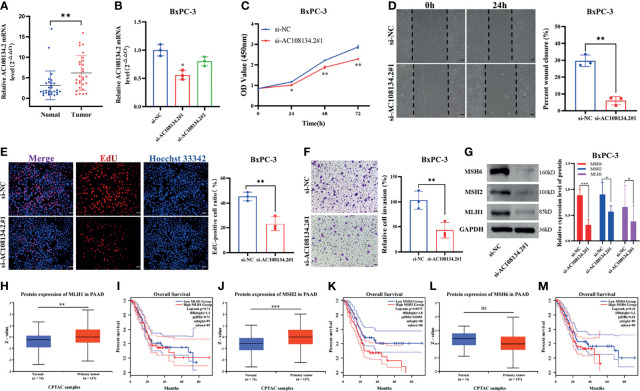
Unfavorable impact of AC108134.2 on PAAD *in vitro*. **(A)** qRT-PCR analysis of AC108134.2 mRNA levels in PAAD tissues and corresponding adjacent tissues (n = 29). **(B)** The knockdown efficiency of AC108134.2 expression in BxPC-3 cells was verified through qRT-PCR. **(C)** CCK-8 assay was used to detect the changes in cell viability of BxPC-3 cells following silencing of AC108134.2. **(D)** Wound healing array was used to analyze the wound healing of BxPC-3 cells downregulated by AC108134.2. **(E)** Changes in the proliferation rate of BxPC-3 cells after silencing of AC108134.2 were analyzed through EdU staining. **(F)** Transwell invasion assay was used to analyze changes in the invasive ability of BxPC-3 cells after silencing of AC108134.2 **(G)** Western blot analysis of the expression of genomic instability-related proteins (MLH1, MSH2, and MSH6) after knockdown of AC108134.2. The CPTAC tool was used to analyze the protein expression levels of MLH1 **(H)**, MSH2 **(J)**, and MSH6 **(L)** in PAAD. GEPIA2.0 server was used to analyze the overall survival rate of MLH1 **(I)**, MSH2 **(K)**, MSH6 **(M)** high, and low expression groups in PAAD.*p < 0.05, **p < 0.01, ***p < 0.001. ns stands for p > 0.05.

In GILncSig, AC108134.2 belongs to the risk gene and is the most critical lncRNA to predict prognosis according to the regression coefficient. Therefore, the present study further evaluated the function of AC108134.2 in PAAD. In 29 PAAD patients collected in the studied hospital, results of the qRT-PCR analysis found that the mRNA level of AC108134.2 was highly expressed in cancer tissues as compared with the corresponding paracancerous tissues (**Figure 8A**, n = 29, *p* = 0.002). To clarify the role of AC108134.2 in PAAD cells, siRNA was transfected into BxPC-3 cells and it was evident that the knockdown efficiency of si-AC108134.2#1 was statistically different (**Figure 8B**, *p* = 0.018). Therefore, si-AC108134.2#1 was used for transfection in subsequent functional experiments.

Results of CCK-8 showed that knockdown of AC108134.2 suppressed the viability of BxPC-3 cells (**Figure 8C**). Wound-healing assays also indicated that AC108134.2 knockdown inhibited PAAD cell migration (**Figure 8D**, *p* = 0.0067). The proliferation rate of BxPC-3 cells was significantly inhibited after downregulation of AC108134.2 as compared with the control group (**Figure 8E**, *p* = 0.0053). Results of the transwell invasion assay also showed that knockdown of AC108134.2 significantly inhibited BxPC-3 cell invasion as compared with the negative control group (**Figure 8F**, *p* = 0.0073). The described results suggest that the expression of AC108134.2 is associated with the proliferation, invasion, and migration of PAAD cells.

The present study also explored how AC108134.2 affects genomic instability. Results of western blotting showed that after the knockdown of AC108134.2, the expression of genomic instability-related proteins (MLH1, MSH2, and MSH6) was decreased (**Figure 8G**). It was also found that when combined with the analysis of the CPTAC data portal and the GEPIA server, the MLH1 and MSH2 proteins were highly expressed in PC tissues (**Figures 8H, J** **)**, and PAAD patients with high MSH2 expression had a significantly lower survival rate (**Figure 8K**). However, there was no statistical difference between MLH1 and overall survival (**Figure 8I**). However, there were no differences in MSH6 protein expression and overall survival (**Figures 8L, M** **)**. In conclusion, the results of the current study suggest that AC108134.2 is associated with genomic instability and that its high expression is associated with PAAD progression.

### Exploring Possible Functions of GILncSig

**Figure 9:**
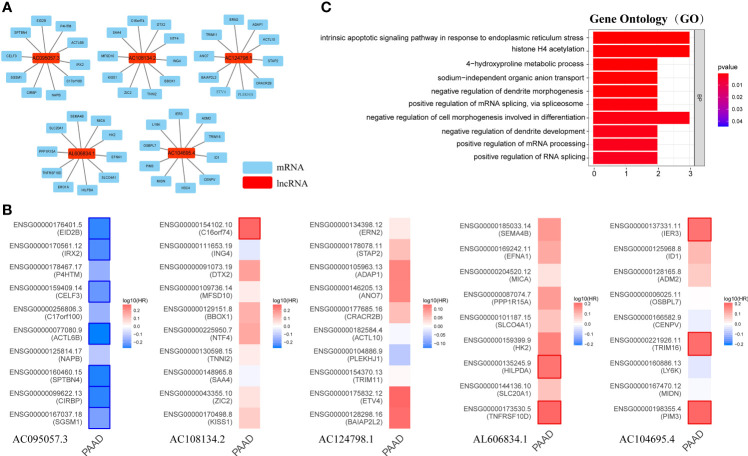
Possible function associated with GILncSig. **(A)** GILncRNA-mRNA co-expression network. **(B)** The GEPIA2 server makes survival maps for the mRNAs related to AC095057.3, AC108134.2, AC124798.1, AL606834.1, and AC104695.4 respectively (Blue and red boxes represent statistically significant differences in survival analysis; Mantel–Cox test). **(C)** GO term enrichment analysis of mRNA co-expressed with GILncRNA. Ten functions that are most relevant to GILncSig were shown through the co-expressed mRNAs enriching in the biological process (BP) of GO.

Long non-coding RNA (LncRNA) does not encode protein. To explore the potential functions of GILncSig, the current study first identified the 10 mRNAs most closely associated with GILncSig through Pearson analysis, and plotted the GILncSig-mRNA co-expression network (**Figure 9A**). The associated mRNAs were then analyzed by the GEPIA2 server to verify the accuracy of the association. It was found that AC095057.3 acted as a protective factor and its associated mRNA was also a protective factor for PAAD, among which EID2B, IRX2, CELF3, ACTL6B, SPTBN4, CIRBP, and SGSM1 were statistically significant mRNAs with better prognosis in PAAD. On the contrary, AC108134.2, AL606834.1, and AC104695.4 were used as risk factors, and their associated mRNAs were also poor prognostic factors in PAAD. The mRNAs with significant statistical significance included C16orf74, HILPDA, TNFRSF10D, IER3, TRIM16, and PIM3 (**Figure 9B**).

Further, GO term enrichment analysis showed that these genes related to GILncSig were mainly enriched in biological process (BP), namely, positive regulation of mRNA splicing through spliceosome, positive regulation of mRNA processing, positive regulation of RNA splicing, negative regulation of cell morphogenesis involved in differentiation, intrinsic apoptotic signaling pathway in response to endoplasmic reticulum stress, and histone H4 acetylation among others (**Figure 9C**). This suggested that GILncSig-related coding genes might be involved in the occurrence and development of GI through the described functions and channels.

### Relationship Between GILncSig and Sensitivity to Chemotherapy

**Figure 10:**
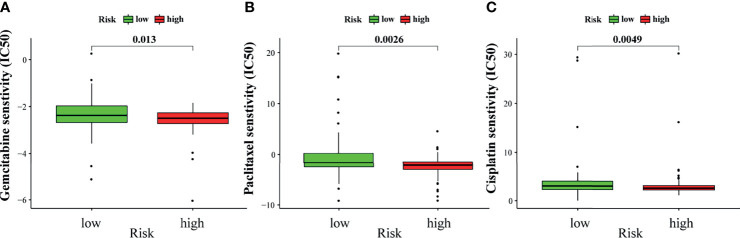
Sensitivity to chemotherapy in high-risk and low-risk groups. The high-risk group was related to a lower IC50 for gemcitabine **(A)**, paclitaxel **(B)**, and cisplatin **(C)**.

Chemotherapy is an important treatment for PAAD. Therefore, the sensitivity of GILncSig to chemotherapeutic agents was also predicted to better guide clinical practice. The IC50 of commonly used chemotherapeutic agents for patients with PAAD in the high- and low-risk groups was calculated and compared through pRRophetic analysis. Gemcitabine, paclitaxel, and cisplatin had low IC50 in the high-risk group (**Figures 10A–C**). This result suggests that patients with higher risk scores are more sensitive to gemcitabine, paclitaxel, and cisplatin, and hence may benefit more from chemotherapy with these drugs.

## Discussion

Pancreatic adenocarcinoma (PAAD) is a malignant disease with persistent GI (39), which is difficult to detect in the early stage. It has a poor treatment effect when diagnosed in the late stage, resulting in a high fatality rate. Pancreatic ductal adenocarcinoma (PDAC) is the most common pathologic type of PAAD and its main treatment is systemic chemotherapy. However, the survival of patients with PDAC is still very limited. Therefore, several new therapeutic approaches are currently emerging, with which targeted therapy alone in combination with standard cytotoxic therapy has been disappointing. Furthermore, integration of genomic analysis and the tumor microenvironment and immunology are found to be contributors to the treatment of PDAC (40). However, tumor immunology therapy still faces great challenges in the clinical application of PDAC (41).

GI and mutation cannot only affect the tumor itself through incorrect DNA replication (42) and DNA damage repair (DDR) (43) among others but also regulate stromal cells and extracellular matrix in direct and indirect ways (40, 44). For example, the first inhibitor of KRAS mutation, AMG 510, has been demonstrated to have good antitumor activity in clinical trials (45). Recently, several studies have proposed the use of single LncRNA as a prognostic marker for patients with PAAD, such as Linc00675 (46), HMlincRNA717 (47), NORAD (48), SNHG15 (49) and NT5E (50). However, the accuracy of prognosis prediction of a single LncRNA needs to be improved. Therefore, a prognostic signature composed of multiple lncRNA biomarkers has received extensive attention from researchers and thus, the current study was aimed to establish a valid and reliable prognostic risk signature based on GI-related LncRNAs for patients with PAAD.

In the modeling process, 409 LncRNAs related to GI were first screened. A total of 171 patients were then randomly divided into the training (n = 86) and testing (n = 85) sets. Univariate and multivariate Cox regression analyses were conducted on the survival data of the training set, and a GILncSig was obtained consisting of five GILncRNAs. The signature was confirmed to have high survival prediction performance through survival analysis, prediction performance test, signature comparison, and other evaluation as well as verification.

Notably, there is has been no previous study that reported the effects of the GILncSig (AC108134.2, AL606834.1, AC104695.4, AC095057.3, and AC124798.1) evaluated on the present study against PAAD but only seemed to point to their effect on other types of cancer. For instance, AC108134.2 is a biomarker for poor prognosis of glioblastoma multiforme (51) whereas AL606834.1 meant poor survival of patients with lung adenocarcinoma (52). It has also been reported that AC104695.4 is closely related to the expression of TGFβ1 in TNBC tissues (53). Moreover, AC095057.3 was also a LncRNA associated with epithelial–mesenchymal transformation, which can be used to predict the overall survival of renal clear cell carcinoma (54). Therefore, this was the first time that GILncRNAs are proposed as the signature for patients with PAAD.

Subsequently, the lncRNA (AC108134.2) was validated with the highest risk factor in GILncSig *in vitro*. Results of the present experimental study showed, for the first time that AC108134.2 is associated with proliferation, migration, and invasion of PAAD cells. It was also found that the involvement of AC108134.2 in PAAD genomic instability may be related to its regulation of MSH2, MSH6, and MLH1 (55–57). In addition, the expression of MLH1, MSH2, and MSH6 proteins in PAAD and their effect on overall survival were analyzed through online databases. It was evident that MSH2 was highly expressed in PAAD and was associated with a poor prognosis. Further, it was evident that the protein expression of MSH2 was downregulated with the knockdown of AC108134.2. Therefore, it can be speculated that AC108134.2 may function by regulating the expression of MSH2 in PAAD. However, there is still a need for further future exploration of its deeper mechanism issues.

Furthermore, in the present study, the top 10 mRNAs associated with GILncSig were found to form LncRNA–mRNA co-expression network and GO enrichment analyses were performed to explore the possible functions of GILncSig. It was found that these GILncSig-related genes were mainly enriched in positive regulation of mRNA splicing through spliceosome, positive regulation of mRNA processing, positive regulation of RNA splicing, negative regulation of cell morphogenesis involved in differentiation, intrinsic apoptotic signaling pathway in response to endoplasmic reticulum stress, and histone H4 acetylation among others which were closely associated with GI.

The RNA processing usually affected the occurrence and progression of PAAD and hence was a key factor of GI. For example, RNA splicing which is the enzymatic process of removing segments of premature RNA to produce mature RNA (58), altered by mutant p53 activates oncogenic RAS signaling in PAAD (36). Upregulation of METTL14 mediates the elevation of PERP mRNA N^6^ adenosine methylation and hence promotes the growth and metastasis of PAAD (59). RNA demethylase ALKBH5 prevents PAAD progression through posttranscriptional activation of PER1 in an m6A-YTHDF2-dependent manner (60). Apoptosis interacted with GI and also promoted the development of PAAD. It was evident that GI, including DNA double-strand breaks (DSBs), telomere dysfunction, illegal polyploidy, and abnormal mitosis, could directly trigger apoptosis through the default pathway. Furthermore, apoptosis that has been suppressed for some reason increased the risk of chromosomal instability (CIN) at different levels and cells that were fit enough to survive may have a growth advantage that leads to cancer (61). In addition, histone H4 acetylation also regulated and controlled GI. For instance, histone H4 acetylation maintains genomic stability by sensing, processing, and repairing the damaged DNA (62). Further, histone H4 acetylation can also inhibit Sir-mediated abnormal aggregation of telomere heterochromatin and promotes telomere transcription, replication, and recombination, hence making telomere plastic and maintaining genome stability (63).

Finally, it was evident that the high-risk group was associated with a decrease in IC50 of the commonly used chemotherapy drugs (gemcitabine, paclitaxel, and cisplatin) against PAAD. This may indicate that the signature has potential predictive properties of chemotherapy sensitivity.

However, the present work also had some shortcomings. First, the sample size used in the study was small. This is mainly due to the limited sample size of PAAD in current public databases. Second, although the performance of the signature was partially validated using GSE102238 and *in vitro* experiments, the ideal signature should also be able to validate the results in another independent cohort. Therefore, there is a need for characterization of the accuracy and precision of the signature in a future multicenter, large sample size cohort.

## Conclusion

In general, the present study evaluated the potential regulatory mechanism, prognostic prediction performance, and chemotherapeutic drug sensitivity of LncRNA related to genome instability. It was evident that the evaluated risk signature has good prognostic performance and may become a potential biomarker that is sensitive to PAAD chemotherapy.

## Data Availability Statement

Publicly available datasets were analyzed in this study. This data can be found in the TCGA database here: https://portal.gdc.cancer.gov/, and the GSE102238: https://www.ncbi.nlm.nih.gov/geo/query/acc.cgi?acc=GSE102238.

## Ethics Statement

The studies involving human participants were reviewed and approved by The Second Affiliated Hospital of Nanchang University Medical Research Ethics Committee. The patients/participants provided their written informed consent to participate in this study.

## Author Contributions

JZ and QH conceived the research and wrote the manuscript. JZ, QH, ZL, and XW downloaded and analyzed the data. XP and SL conducted the experiments. HL participated in processing the data and guided the study. HL, XP, and CL made manuscript revisions and approved the final draft. All authors listed have made a substantial, direct, and intellectual contribution to the work and approved it for publication.

## Conflict of Interest

The authors declare that the research was conducted in the absence of any commercial or financial relationships that could be construed as a potential conflict of interest.

## Publisher’s Note

All claims expressed in this article are solely those of the authors and do not necessarily represent those of their affiliated organizations, or those of the publisher, the editors and the reviewers. Any product that may be evaluated in this article, or claim that may be made by its manufacturer, is not guaranteed or endorsed by the publisher.
